# Writer’s Cramp

**Published:** 1882-01

**Authors:** 


### WRITER'S CRAMP.

This and allied defects from over use of certain muscles, are more successfully treated by galvanism than by any other means. With galvanism must be conjoined rest and systematic gymnastic training. Indeed, without rest, no improvement can take place in the condition of the affected muscles. The state of the muscles in writer's cramp varies in different cases. There may be cramps of the muscles concerned in the prehension of the pen; there may be a condition of fatigue and exhaustion, or some of the muscles may be paretic. Some of the cases are local and muscular; some are local and nervous, and a small proportion have their origin in intra-cranial lesions, in changes in the motor and co-ordinating centres. It is obvious that the treatment must be adapted to the conditions present. As most of the cases are due to muscular fatigue and cramps, the most appropriate remedy is galvanism, but this must be conjoined with rest, massage and gymnastics. The anode should be placed over the cervical plexus, and the cathode brushed over the muscular groups in turn, from the shoulders down. If the defect is confined to the thumb and finger muscles, to the thenar group, the interossei, and flexors of the fingers, the applications should rather be confined to those parts and consist in the descending labile current. If the lesion consists in relaxation, paresis and degeneration of any of the muscles, faradism may then be employed with advantage. Duchenne's electrodes are best adapted to cases requiring application to individual muscles. Those affected must be selected out, and a current of strength necessary to induce contractions merely, passed through them. Under no circumstances ought the muscles be tired, either by the strength or duration of the applications. Treated in accordance with these principles, recent cases of writer's cramp may be cured or ameliorated.—*Bartholow's Medical Electricity. Maryland Med. Jour., June 1st. Braithwaite's Retrospect.*

---

Many dentists seem to forget that if they consent to operate for a patient, whether there is a remunerative consideration involved or none at all, they are under moral obligations to do the best possible for that patient.

